# Declining Incidence of Hepatitis C Virus Infection among People Who Inject Drugs in a Canadian Setting, 1996-2012

**DOI:** 10.1371/journal.pone.0097726

**Published:** 2014-06-04

**Authors:** Jason Grebely, Viviane Dias Lima, Brandon D. L. Marshall, M-J Milloy, Kora DeBeck, Julio Montaner, Annick Simo, Mel Krajden, Gregory J. Dore, Thomas Kerr, Evan Wood

**Affiliations:** 1 The Kirby Institute, UNSW Australia, Sydney, New South Wales, Australia; 2 British Columbia Centre for Excellence in HIV/AIDS, Vancouver, British Columbia, Canada; 3 Division of AIDS, Department of Medicine, Faculty of Medicine, University of British Columbia, Vancouver, British Columbia, Canada; 4 Department of Epidemiology, Brown University, Providence, Rhode Island, United States of America; 5 Department of Family Practice, Faculty of Medicine, University of British Columbia, Vancouver, British Columbia, Canada; 6 School of Public Policy, Simon Fraser University, Vancouver, British Columbia, Canada; 7 British Columbia Centre for Disease Control, Vancouver, British Columbia, Canada; University of Washington, United States of America

## Abstract

**Background:**

People who inject drugs (PWID) are at high risk of hepatitis C virus (HCV) infection. Trends in HCV incidence and associated risk factors among PWID recruited between 1996 and 2012 in Vancouver, Canada were evaluated.

**Methods:**

Data were derived from a long-term cohort of PWID in Vancouver. Trends in HCV incidence were evaluated. Factors associated with time to HCV infection were assessed using Cox proportional hazards regression.

**Results:**

Among 2,589, 82% (n = 2,121) were HCV antibody-positive at enrollment. Among 364 HCV antibody-negative participants with recent (last 30 days) injecting at enrollment, 126 HCV seroconversions were observed [Overall HCV incidence density: 8.6 cases/100 person-years (py); 95% confidence interval (95% CI): 7.2, 10.1; HCV incidence density among those with injecting during follow-up: 11.5 cases/100 py; 95% CI 9.7, 13.6]. The overall HCV incidence density declined significantly from 25.0/100 py (95% CI: 20.2, 30.3) in 1996–99, as compared to 6.0/100 py (95% CI: 4.1, 8.5) in 2000–2005, and 3.1/100 py (95% CI: 2.0, 4.8) in 2006–2012. Among those with injecting during follow-up, the overall HCV incidence density declined significantly from 27.9/100 py (95% CI: 22.6, 33.6) in 1996–99, as compared to 7.5/100 py (95% CI: 5.1, 10.6) in 2000–2005, and 4.9/100 py (95% CI: 3.1, 7.4) in 2006–2012. Unstable housing, HIV infection, and injecting of cocaine, heroin and methamphetamine were independently associated with HCV seroconversion.

**Conclusions:**

HCV incidence has dramatically declined among PWID in this setting. However, improved public health strategies to prevent and treat HCV are urgently required to reduce HCV-associated morbidity and mortality.

## Introduction

Hepatitis C virus (HCV) infection remains a considerable health problem among people who inject drugs (PWID). Globally, it is estimated that 10 million PWID are HCV-infected worldwide, corresponding to a mid-point prevalence of 67% [1]. HCV transmission continues to occur among PWID, with most estimates of HCV incidence in this group ranging from 10 to 40 cases per 100 person-years [2]–[18]. However, several studies have indicated that HCV incidence among PWID may be decreasing in some settings [2], [8], [9]. Understanding the long-term trends and factors associated with HCV incidence is crucial for the development and evaluation of HCV prevention and treatment programs for PWID.

Beginning in 1994, Vancouver, Canada experienced an outbreak of HCV infection among PWID, with an incidence of 29 per 100 person-years reported between 1996 and 1999 [4]. Factors independently associated with HCV seroconversion included female sex and at least daily injecting drug use, specifically cocaine injecting [4]. Over the past 15 years, there have been substantial changes in Vancouver’s drug markets and an increased availability of services intended to prevent HCV and human immunodeficiency virus (HIV) infections [including the expansion of needle and syringe programs (NSP), opioid agonist therapy (OAT) and the establishment of a medically supervised safer injecting facility (Insite)]. A decrease in the incidence of HCV infection has been observed at the population-level in the Province of British Columbia [19], but little is known about the trends in HCV incidence among PWID in this setting. Therefore, the aim of this study was to investigate trends in HCV incidence and factors associated with HCV infection among a cohort of PWID in Vancouver, Canada from 1996 to 2012.

## Methods

### Study Population and Design

The Vancouver Injection Drug Users Study (VIDUS) is an open prospective community-recruited cohort of PWID in Vancouver, Canada. Beginning in May 1996, active PWID (i.e. those who reported injecting drugs in the previous month) were recruited in the Greater Vancouver region on an ongoing basis throughout the study period. Recruitment strategies employ extensive street-based outreach and “snowball” sampling approaches. Given VIDUS is an open cohort, new participants were continuously enrolled in the cohort over the study period to replace those who died or were lost to follow-up. All participants were recruited through street outreach, word of mouth, and self-referral, and provided written informed consent prior to entering the study. The University of British Columbia/Providence Health Care Research Ethics Board approved this study.

For the current study, all participants who completed a baseline survey between May 1996 and December 2012 were eligible for inclusion. Individuals at-risk for HCV infection included participants who were HCV antibody negative at the study enrolment visit and had at least one follow-up visit. Analyses were performed among the overall study population (those who reported injecting in the previous month at cohort entry) and among those who reported injecting during follow-up (previous six months) to assess outcomes specifically among those injecting during the study period. Three study periods were identified *a priori* to control for potential cohort effects (1996–99, 2000–2005, and 2006–2012). These periods were chosen based on the fact that between 2000 and 2002 there were substantial changes by the Vancouver health authority to move from syringe exchange to syringe distribution to maximize access to sterile syringe access, which was associated with reduced rates of syringe sharing among PWID in Vancouver [20]. As such, these periods represented a pre-intervention (1996–1999), intermediate intervention (2000–2005) and post-intervention period (2006–2012).

### Study Assessments

At baseline and semi-annually, participants completed a harmonized interviewer-administered questionnaire. Sociodemographic data, as well as information pertaining to drug use patterns, risk behaviors, and health care utilization were collected. The survey for each study consisted of a uniform set of questions, which permitted the aggregation and analysis of data from all enrolled participants. Nurses collected blood samples for HIV and hepatitis C serology, and also provided basic medical care and referrals to appropriate health care services. Participants received $20 for each study visit.

### Measurements

The primary study outcome was HCV seroconversion (defined by an HCV antibody negative test at enrolment followed by an HCV antibody positive test). The estimated date of HCV seroconversion was calculated as the mid-point between the last negative and first positive HCV antibody test. For those with HCV seroconversion, follow-up time was calculated from the first HCV antibody negative test until the estimated date of HCV seroconversion. For those without HCV seroconversion, follow-up time was calculated from the first to the last HCV antibody negative test observed during the study period.

### Statistical Analyses

Descriptive analyses were performed to characterize the study population during three enrolment periods (1996–1999, 2000–2005 and 2006–2012). The characteristics of participants enrolled across these three enrolment periods were compared using Fisher’s exact test for categorical variables and the Kruskal-Wallis test for continuous variables. To test for trends across enrolment periods, the Cochran-Armitage trend test was used for categorical variables and linear regression for continuous variables (e.g., age).

The HCV incidence density and confidence intervals were calculated by the person-years method among those who were negative for HCV antibodies at study enrolment among: i) the overall study population (those who reported injecting in the previous month at cohort entry); and ii) among those who contributed injecting person-years during follow-up (previous six months). Trends in HCV incidence density were assessed by calendar year, calendar period (1996–1999, 2000–2005, and 2006–2012), enrolment period (1996–1999, 2000–2005, and 2006–2012) and a combination of both enrolment period and calendar period. This was also presented visually using a diamond graph, which shows a continuous outcome across two categorical strata [21].

Cox proportional hazards analyses were used to identify factors associated with time to HCV seroconversion among the overall study population (those who reported injecting in the previous month at cohort entry), allowing for time-dependent variation in injecting behaviors during follow-up. Hypothesized factors associated with HCV incidence were determined *a priori* and included the following baseline characteristics: sex (male vs. female) [4]; education, defined as high school completion (yes vs. no) [17]; and year of enrolment (1996–1999, 2000–2005, and 2006–2012). Exposures that were measured at baseline and repeatedly during follow-up were treated as time-dependent variables in all survival models and included: age (per year older) [22], [23]; unstable housing status, defined as living in a single occupancy room in a hotel, a treatment or recovery house, jail, shelter or hostel, or having no fixed address in the last 6 months (yes vs. no); unemployment in the last 6 months (yes vs. no); various measures of drug use, including crack cocaine use via smoking, any injecting drug use, injecting heroin, injecting cocaine and injecting methamphetamine (including crystal methamphetamine) in the last 6 months [4], [22], [24], [25]; syringe borrowing, defined as injecting with a used syringe in the last 6 months [22]; and HIV status.

Two separate models were evaluated for each population of interest including: i) one model considering any injection drug use (i.e., without injecting cocaine, heroin or methamphetamine entered into the model); and ii) one model with each type of injecting drug entered separately (two separate models in total). The selection of variables for the multivariable models was based on two criteria: Akaike Information Criterion (AIC) and Type III p-values, as has previously been described [26], [27]. Briefly, these two criteria balance the model choice on finding the best explanatory model (Type III p-values – lower p-values indicate more significance) and at the same time a model with the best goodness-of-fit statistic (AIC – lower values indicate better fit). At each step of this process, the AIC value and the Type III p-values of each variable are recorded, and the variable with the highest Type III p-value is dropped, until there are no more variables left in the model. The final model has the lowest AIC. Statistically significant differences were assessed at *p*<0.05; p-values are two-sided. All analyses were performed using SAS software (version 9.2; SAS Institute Inc., Cary, North Carolina, USA).

## Results

### Participant Characteristics

**Figure 1:**
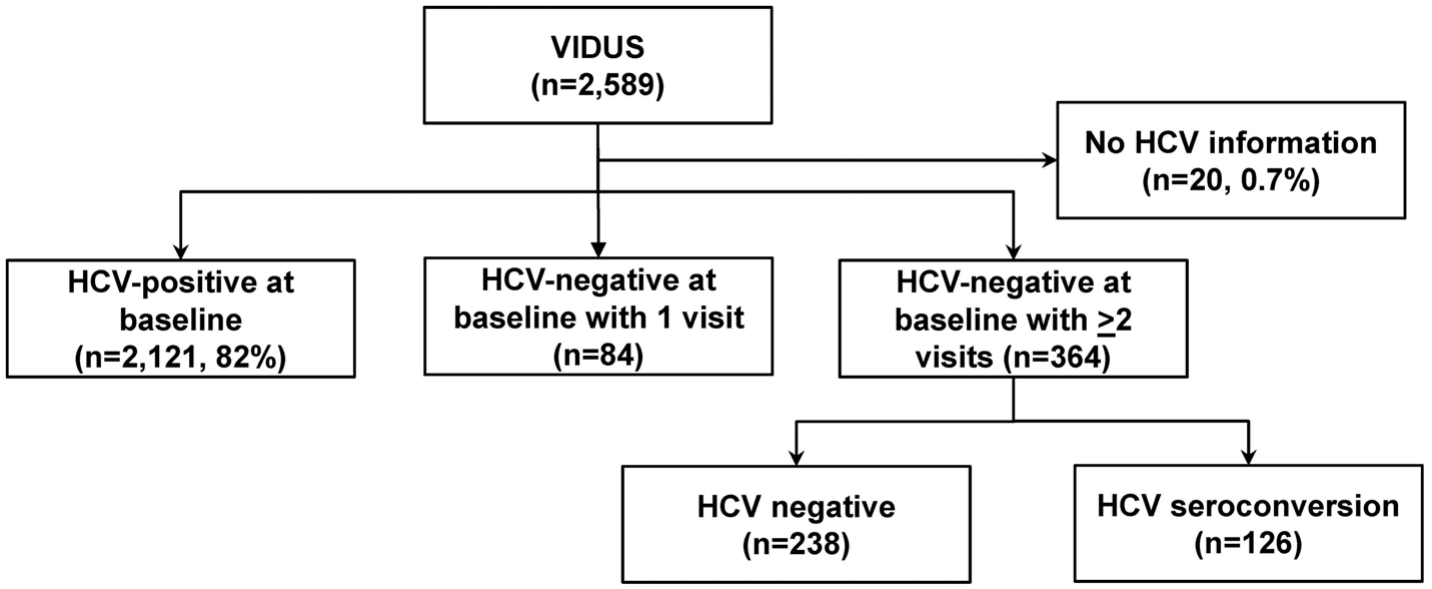
Participant disposition.

In total, 2,589 unique participants were eligible for inclusion in this analysis (Figure 1). At enrolment, 82% (2,121 of 2,589) were HCV antibody positive. Among participants who were HCV antibody negative at enrolment (n = 448), 364 participants had ≥2 visits and were eligible for the assessment of trends in HCV incidence. Compared to those with no follow-up visits, those with follow-up and included in the analysis were more likely to be older (Table S1). There were no other significant differences in sociodemographic characteristics between those with and without follow-up.

**Figure 2:**
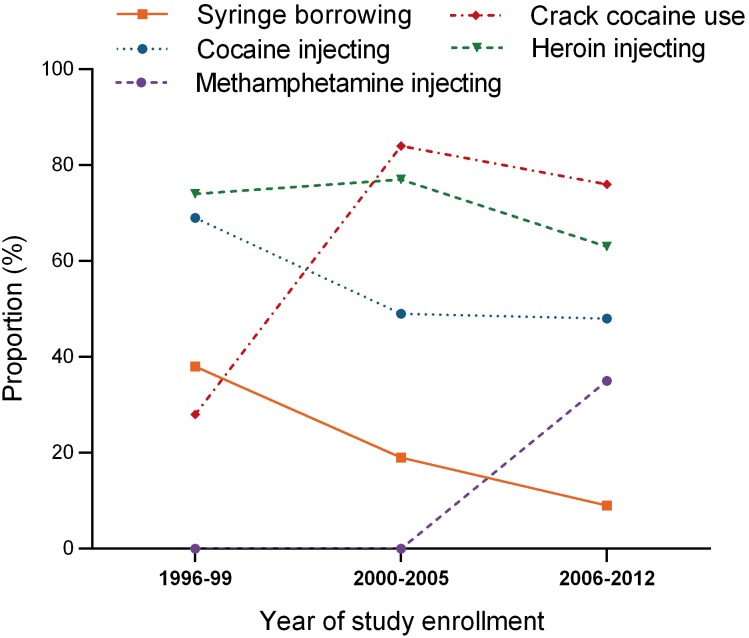
Trends in syringe borrowing and drug use at enrolment among HCV antibody negative PWID enrolled in the VIDUS cohort in Vancouver, Canada by year of enrollment.

Table 1 illustrates the characteristics of the cohort separated into three enrolment periods (1996–99, 2000–2005, and 2006–2012). Compared with the 1996–1999 cohort, the 2006–2012 cohort was older, had a lower proportion of females and a greater proportion with a high school education or higher. The proportion of participants reporting any syringe borrowing in the last 6 months and injecting cocaine use decreased over time (Table 1 and Figure 2). Crack cocaine and injecting methamphetamine use at the time of study enrollment increased over time (Table 1 and Figure 2). The proportion reporting injecting heroin remained stable over time (Figure 2).

**Table 1:** Characteristics of HCV antibody negative PWID by year of enrolment in the VIDUS cohort between 1996 and 2012 in Vancouver, Canada (n = 364).

	Year of Enrollment				
Variable	1996–1999(n = 198) n (%)	2000–2005(n = 69) n (%)	2006–2012 (n = 97) n (%)	*P* ¶	*P* §
Median age (25–75^th^ percentiles)*	25 (20–35)	23 (21–26)	39 (31–47)	<0.001	<0.001
Female sex	63 (32%)	32 (46%)	20 (21%)	0.002	0.144
High school education or higher*	45 (23%)	19 (28%)	40 (45%)	<0.001	<0.001
Unstable housing†	122 (62%)	44 (64%)	61 (64%)	0.924	0.724
HIV infection†	10 (5%)	1 (1%)	26 (27%)	<0.001	<0.001
Crack cocaine use (smoking)†	56 (28%)	58 (84%)	74 (76%)	<0.001	<0.001
Syringe borrowing†	74 (38%)	13 (19%)	9 (9%)	<0.001	<0.001
Cocaine Injecting†	137 (69%)	34 (49%)	47 (48%)	<0.001	<0.001
Heroin Injecting†	147 (74%)	53 (77%)	61 (63%)	0.083	0.065
Methamphetamine injecting†	0 (0%)	0 (0%)	34 (35%)	<0.001	<0.001

Percentages indicate column percentages;

*At the time of enrolment;

† in the last 6 months prior to enrolment;

¶ p-value for association (Fisher’s exact test for categorical variables, Kruskal-Wallis Test for continuous variables);

§ p-value for trend (Cochran-Armitage test for categorical variables, linear regression for continuous variables).

### Trends in HCV Incidence Density

**Figure 3:**
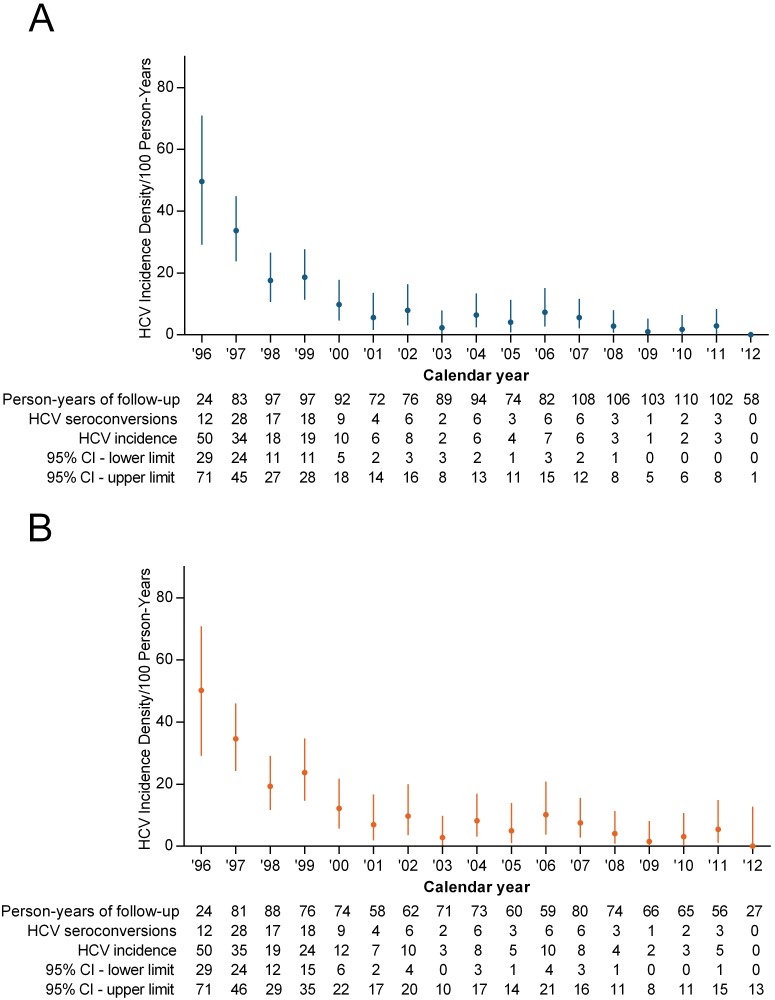
Annual incidence density of HCV infection (per 100 person-years) among PWID in the VIDUS cohort between 1996 and 2012. A) overall population (injecting at enrolment); B) those with person-years of injecting during follow-up. The circles indicate the HCV incidence density (per 100 person years) and the lines indicate the 95% confidence intervals (95% CI).

Among 364 participants overall who were HCV antibody negative at enrollment with ≥2 visits, 126 HCV seroconversions were observed between 1996 and 2012 over 1,466 person-years (py) of follow-up, resulting in an overall HCV incidence density of 8.6 cases/100 py [95% confidence interval (95% CI): 7.2, 10.1]. The average number of study visits was 7.6 and the median (Q1–Q3) was 5.0 (2.0–10.5). The average follow-up (in years) was 4 years and the median (Q1–Q3) was 2.8 (0.9–6.0) years. The annual HCV incidence density by calendar year of observation is shown in Figure 3A.

When these analyses were restricted to HCV antibody negative participants with recent injecting during follow-up, 126 HCV seroconversions were observed between 1996 and 2012 over 1,094 py of follow-up, resulting in an overall HCV incidence density of 11.5 cases/100 py [95% confidence interval (95% CI): 9.7, 13.6]. The annual HCV incidence density by calendar year of observation in those with injecting person-years of follow-up is shown in Figure 3B.

**Figure 4:**
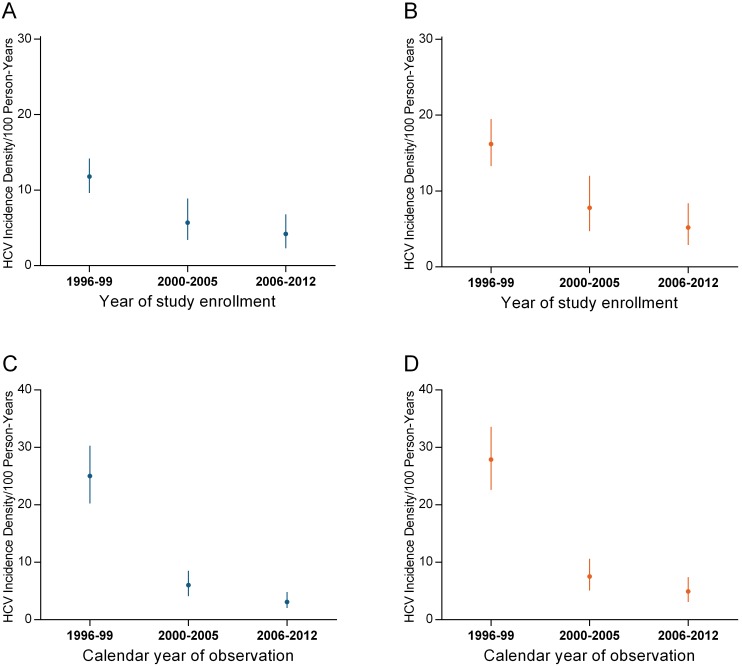
Incidence density of HCV infection (per 100 person-years) among PWID in the VIDUS cohort by year of study enrollment in: A) the overall population; B) those with person-years of injecting during follow-up; and calendar year of observation in: C) the overall population; and D) those with person-years of injecting during follow-up. The circles indicate the HCV incidence density (per 100 person years) and the lines indicate the 95% confidence intervals (95% CI).

As shown in Figure 4A, by calendar year of enrolment, the HCV incidence density declined significantly from 11.8/100 py (95% CI: 9.6, 14.2) in 1996–99, as compared to 5.7 cases/100 py (95% CI: 3.4, 8.9) in 2000–2005, and 4.2 cases/100 py (95% CI: 2.3, 6.8) in 2006–2012. As shown by Figure 4B, by calendar year of observation, the HCV incidence density declined significantly from 25.0/100 py (95% CI: 20.2, 30.3) in 1996–99, as compared to 6.0 cases/100 py (95% CI: 4.1, 8.5) in 2000–2005, and 3.1 cases/100 py (95% CI: 2.0, 4.8) in 2006–2012.

As shown in Figure 4C, when these analyses were restricted to HCV antibody negative participants with recent injecting during follow-up, the HCV incidence density declined significantly by calendar year of enrolment, from 16.2/100 py (95% CI: 13.3, 19.5) in 1996–99, as compared to 7.8 cases/100 py (95% CI: 4.7, 12.0) in 2000–2005, and 5.2 cases/100 py (95% CI: 2.9, 8.4) in 2006–2012. As shown in Figure 4D, when these analyses were restricted to HCV antibody negative participants with recent injecting during follow-up, the HCV incidence density declined significantly by calendar year of observation, from 27.9/100 py (95% CI: 22.6, 33.6) in 1996–99, as compared to 7.5 cases/100 py (95% CI: 5.1, 10.6) in 2000–2005, and 4.9 cases/100 py (95% CI: 3.1, 7.4) in 2006–2012.

**Figure 5:**
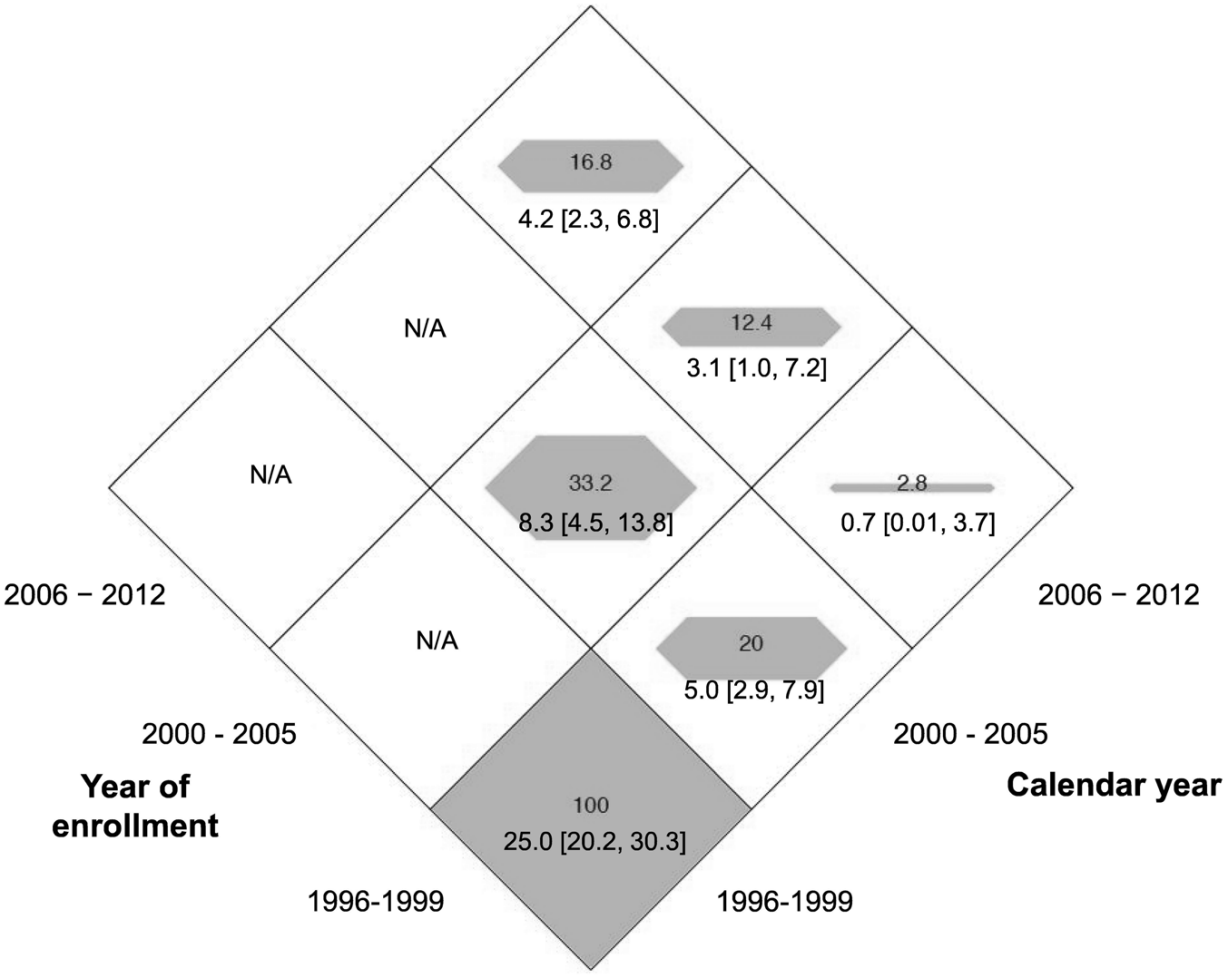
Incidence density of HCV infection (per 100 person-years) among PWID in the VIDUS cohort by calendar year of enrolment and calendar year of observation. Each shaded area is a proportion relative to the largest incidence rate with 25.0 being 1 (full shading). The numeric values of the rates are used as the labels [95% CI]. Cells with N/A are blank.

As shown in Figure 5, trends in HCV incidence density were assessed by a combination of both enrolment period and calendar period of observation. Among those enrolled in 1996–1999 (Figure 5), the HCV incidence density declined from 25.0/100 py (95% CI: 20.2, 30.3) in 1996–99, as compared to 5.0 cases/100 py (95% CI: 2.9, 7.9) in 2000–2005, and 0.7 cases/100 py (95% CI: 0.01, 3.7) in 2006–2012. Among those enrolled in 2000–2005, the HCV incidence density was 8.3/100 py (95% CI: 4.5, 13.8) in 2000–2005, and 3.1 cases/100 py (95% CI: 1.0, 7.2) in 2006–2012. Among those enrolled in 2006–2012, the HCV incidence density was 4.2/100 py (95% CI: 2.3, 6.8) in 2006–2012.

### Factors Associated with HCV Seroconversion

**Figure 6:**
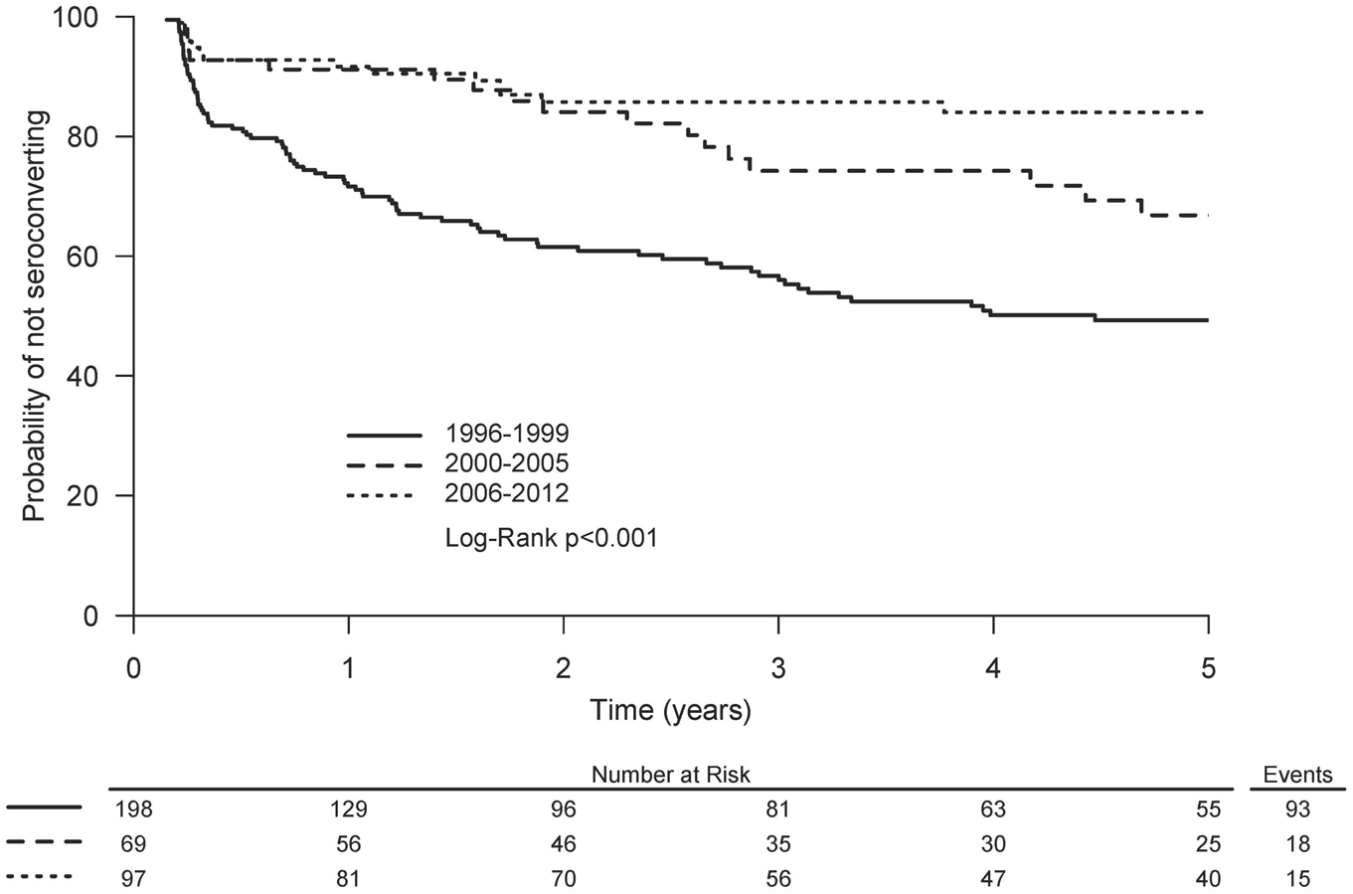
Kaplan-Meier graphs of time to HCV seroconversion by calendar year of enrolment between 1996 and 2012 among PWID in the VIDUS cohort (truncated at 5 years of follow-up).

Factors associated with HCV seroconversion in unadjusted Cox proportional hazards analyses are shown in Table 2. In both Kaplan-Meier analyses (Figure 6 and Figure S1) and unadjusted Cox proportional hazards analyses (Table 2), enrolment periods 2000–2005 and 2006–2012 were associated with reduced HCV seroconversion. In adjusted Cox proportional hazards analyses in the overall study population, HCV infection was associated with older age, unstable housing, HIV infection, cocaine injecting, heroin injecting and methamphetamine injecting (Table 2). The enrolment periods 2000–2005 and 2006–2012 were also associated with reduced HCV seroconversion.

**Table 2:** Cox proportional hazards analysis of factors associated with time to HCV seroconversion among PWID in the VIDUS cohort between 1996 and 2012 in Vancouver, Canada.

Variables	Unadjusted HR	Model 1 Adjusted for any injecting drugs AHR¥ (95% CI)	Model 2 Adjusted for specific injecting drugs AHR‡ (95% CI)
*Fixed*			
**Female sex (vs. male sex)**	1.42 (0.95, 2.10)	1.42 (0.95, 2.10)	1.41 (0.95, 2.09)
**High school education or higher (vs. less than high school)** *	0.63 (0.47, 0.85)	-	-
**Year of Enrollment**			
1996–1999	1 (−)	1 (−)	1 (−)
2000–2005	0.76 (0.51, 1.13)	0.49 (0.29, 0.82)	0.49 (0.29, 0.83)
2006–2012	0.21 (0.14, 0.30)	0.21 (0.12, 0.39)	0.17 (0.09, 0.35)
*Time-varying*			
**Age**	1.01 (1.00, 1.03)	1.02 (1.00, 1.04)	1.02 (0.99, 1.04)
**HIV infection (vs. none)** †	0.96 (0.57, 1.60)	1.77 (1.01, 3.10)	1.76 (1.00, 3.11)
**Unstable housing (vs. stable)** †	1.66 (1.22, 2.28)	1.68 (1.15, 2.44)	1.61 (1.10, 2.36)
**Crack cocaine use (smoking) (vs. none)** †	0.78 (0.58, 1.04)	-	-
**Any injection drug use (vs. none)** †	8.43 (5.53, 12.85)	3.42 (1.58, 7.41)	Not included
**Syringe borrowing (vs. none)** †	3.67 (2.66, 5.08)	1.37 (0.91, 2.04)	1.32 (0.88, 1.98)
**Cocaine injecting (vs. none)** †	4.04 (3.03, 5.38)	Not included	1.77 (1.20, 2.61)
**Heroin injecting (vs. none)** †	4.39 (3.26, 5.93)	Not included	1.57 (1.05, 2.35)
**Methamphetamine injecting (vs. none)** †	2.45 (1.74, 3.46)	Not included	2.53 (1.11, 5.73)

HR = hazard ratio;

*At the time of enrolment,

† In the past 6 months,

¥ Model 1 was assessed in the overall population and selected to assess the impact of any injecting drugs and so cocaine injecting, heroin injecting and methamphetamine injecting are not included;

‡ Model 2 was assessed in the overall population and selected to assess the impact of the specific contribution of cocaine injecting, heroin injecting and methamphetamine injecting and so any injecting was not included.

## Discussion

This study characterizes trends in HCV incidence and associated factors among a cohort of people who inject drugs (PWID) recruited between 1996 and 2012 in Vancouver, Canada. HCV incidence among PWID in this cohort has decreased significantly since 1996. The decrease in HCV incidence between 1996 and 2012 was associated with shifts in drug use in the region, characterized by decreases in syringe borrowing, as well as an increase in crack cocaine use. In survival analysis, factors associated with HCV infection included unstable housing, HIV infection, and injecting of cocaine, heroin and methamphetamine. This study provides important insights into the long-term trends in HCV incidence among PWID in Vancouver and highlights factors important for the control of HCV infection among PWID. This information is crucial for the development and evaluation of prevention programs targeted to this group, particularly in settings where HCV incidence remains high in this population.

The incidence of HCV infection declined substantially during the study period, consistent with an overall decrease in HCV incidence at the population-level in the province of British Columbia [19]. Further, people enrolled in 2000–2005 and 2006–2012 had a reduced risk of HCV infection, after adjusting for other factors associated with HCV infection. Although there are few recent international studies following sufficient numbers of PWID over such a long period to assess long-term trends in HCV incidence, this is consistent with the results observed elsewhere. In a long-term cohort of PWID in Amsterdam, the Netherlands, HCV incidence decreased from 27.5/100 py in 1985 as compared to 2.0/100 py in 2004–2005 [2]. In Australia, among PWID attending needle and syringe programs, HCV incidence decreased from a peak of 30.8/100 py in 2003 to a low of 4.0/100 py in 2009 [9]. In a community-based cohort of PWID in Baltimore, United States, a non-significant decline in HCV incidence was observed from 22.0 cases/100 py in 1988–1989 to 7.8 cases/100 py in 2005–2008 [8]. However, HCV incidence remains high in a number of international settings, ranging from 7 to 47/100 py [3], [5]–[8], [10]–[17], and strategies to prevent infection are still urgently needed.

Consistent with previous studies, unstable housing [17], injecting cocaine use [4], [5], [9], [11], [14], [16], [17] and injecting heroin use [9], [11], [17] were associated with HCV infection in this study. Injecting methamphetamine use was also associated with HCV infection, which is a concerning finding, given an increase in methamphetamine use and reports of ready access to methamphetamine among young people who use drugs in Vancouver [28]. Further prevention efforts are needed specifically targeted to methamphetamine injectors, given the increased risk of HCV seroconversion in this group.

Prevention of HCV infection can occur through both reductions in injecting risk behaviours and prevention of initiation into injecting [29]. Mathematical modeling of the impact of NSP on HCV transmission among PWID in Australia has demonstrated that the number of times each syringe is used before disposal is the most sensitive behavioral factor in determining the incidence of HCV infection, followed by the percentage of injections that are shared [30]. In Amsterdam, the Netherlands, modelling suggests that the marked decreases in HIV and HCV among PWID in Amsterdam since 1990 could be due partly to harm reduction measures; however, they may also be attributable to behavior changes in the PWID population (namely reduced injecting drug use) [31]. In Australia, changes to Australian drug markets (heroin shortage in the early 2000s) and the wide-spread availability of NSP and OAT have likely contributed to a decrease in the observed HCV incidence among PWID [9].

In this study, the proportion of HCV antibody negative PWID reporting recent syringe borrowing decreased significantly between 1996–99 and 2006–2012. The observed decrease in syringe sharing is also concurrent with an increase in injecting cessation that has been described in Vancouver over the past decade [32]. Further, in this study, the proportion smoking crack cocaine significantly increased between 1996–99 and 2006–2012, while the proportion reporting any syringe borrowing decreased. The observed increase in crack cocaine smoking is consistent with previous data demonstrating that crack cocaine smoking among PWID in Vancouver has increased substantially, with frequent cocaine injecting and methamphetamine injecting being independent predictors of crack cocaine initiation [33]. Although the inverse association between crack cocaine smoking and HCV infection in this study was not statistically significant in adjusted models, it is plausible that the shift from injecting cocaine to smoking crack cocaine use has resulted in transitions out of injecting, as well as a potentially reduced rate of entry into injecting and, by consequence, lowered risk of HCV infection. Decreased HCV incidence in Vancouver is likely due to a combination of factors, but likely includes decreases in syringe borrowing and decreased entry into injecting drug use (namely, cocaine and heroin). Another key factor is the extremely high baseline HCV prevalence, which was 82% at study entry, and which reduced the number of individuals eligible for subsequent HCV infection during follow-up. Overall, it is important to note that the association between year of enrolment and HCV seroconversion persisted after adjustment for a number of other factors, suggesting that the decline in HCV incidence is not fully explained by these variables and therefore other unmeasured factors may have played a role (e.g. other harm reduction interventions, such as NSP or OST programs).

This study has a number of limitations. The VIDUS cohort is not a random sample of the eligible population of PWID in Vancouver. While analyses indicate that the VIDUS cohort is representative of PWID in the DTES community [34], findings from the present study should be generalized and interpreted with caution. As a result, the findings may not be generalizable to all Vancouver PWID and other urban or remote/rural settings where drug use is common. Information on all behaviours were collected by self-report and may be subject to response biases. Twenty-five percent of participants who were HCV negative were lost-to follow-up, which is also a limitation. This is important because Cox models assume non-informative censoring (lost to follow up is random for exposures and covariates related to the outcome of interest). NSP coverage was not assessed in this study, given previous studies highlighting selection effects involving high-risk participants reporting regular NSP use in this setting [4], [35]. Injecting equipment borrowing was also not assessed in this study, but could be hypothesized to also be associated with HCV infection, given that the risk of HCV infection through shared drug preparation equipment is similar to that of shared syringes [36]. Another potential confounder is left truncation bias, where more recent enrollees may not have been followed for a sufficient time to demonstrate HCV seroconversion. The effect of this would be that as time goes on, early recruits who remain seronegative (e.g. “survivors” whose risk may diminish because of unmeasured factors) will be increasingly over-represented and contribute more person time, thus diluting incidence rates in later calendar periods. In an effort to reduce the impact of left truncation bias, we have combined the later calendar years in subsequent analyses. A problem that is also common among closed cohort studies is that some people may exit the HCV-susceptible cohort due to seroconversion, leading to a cohort with a higher proportion of low-risk individuals, also known as “the depletion of susceptibles”. While it is possible that the observed decreases could partly be attributed to the removal of higher risk individuals, VIDUS is an open cohort and new participants were continuously enrolled in the cohort over the study period to replace those who died or were lost to follow-up. To minimize this potential limitation, analyses were performed to provide estimates of HCV incidence by both enrolment period and calendar year of observation, clearly demonstrating a decreasing HCV incidence over time in this cohort. Further, the Kaplan-Meier of time to HCV seroconversion by calendar year of enrolment demonstrates that newly recruited participants are definitely still at risk of HCV seroconversion, albeit at a lower rate than the older cohorts. Lastly, we cannot exclude the possibility that sampling may have changed during follow-up, leading to a cohort at lower risk of HCV infection over time, despite that the sampling methodologies for VIDUS have remained consistent over the past two decades.

In summary, we have demonstrated a dramatic reduction in the incidence of HCV infection among PWID in the VIDUS study between 1996 and 2012 in Vancouver, Canada. Decreases in HCV incidence were accompanied by increases in crack cocaine smoking and decreases in syringe borrowing coinciding with a scale up of harm reduction programming. Factors independently associated with HCV infection included unstable housing, HIV infection and injecting drug use behaviours. Although the observed declines in HCV incidence are encouraging, we note that HCV transmission continues to occur among PWID in Vancouver.

Further work is needed to better understand HCV transmission pathways among those transmitting and at risk for HCV infection. For example, it is now possible to identify transmission clusters and transmission cluster characteristics through recent advances in molecular epidemiology, phylogenetic, and phylodynamic methodologies [37]. Identifying characteristics of people at high risk of HCV transmission may provide important information for the design and implementation of public health strategies targeted towards PWID in an effort to reduce or eliminate HCV in this population, particularly in settings where HCV incidence remains high. Continued surveillance to monitor trends in drug use, HCV incidence, and risk behaviors is also required. Development and implementation of harm-reduction strategies such as NSP, OAT and other forms of addictions treatment (e.g., methamphetamine injectors) including broader coverage, enhanced early access, and intensification all need to be part of a multi-faceted approach to reduce or eliminate HCV among PWID. Lastly, expanding HCV screening, assessment and treatment among PWID is needed to reduce the HCV-related morbidity and mortality, particularly given the potential prevention benefits of HCV treatment in reducing transmission [38].

## Supporting Information

Figure S1 **Kaplan-Meier graphs of time to HCV seroconversion by calendar year of enrolment among PWID in the VIDUS cohort between 1996 and 2012 in Vancouver, Canada.**
(TIF)

Table S1 **Participant enrolment characteristics among HCV antibody negative participants with no follow-up visits (1 visit) and follow-up visits (≥2 visits) enrolled in the VIDUS cohort in Vancouver, Canada between 1996 and 2012.**
(DOCX)
