# Supplementary figures and images for: Declining Incidence of Hepatitis C Virus Infection among People Who Inject Drugs in a Canadian Setting, 1996-2012
Source: PLoS One. 2014 Jun 4;9(6):e97726. doi: 10.1371/journal.pone.0097726 (PMC4045728; doi:10.1371/journal.pone.0097726)

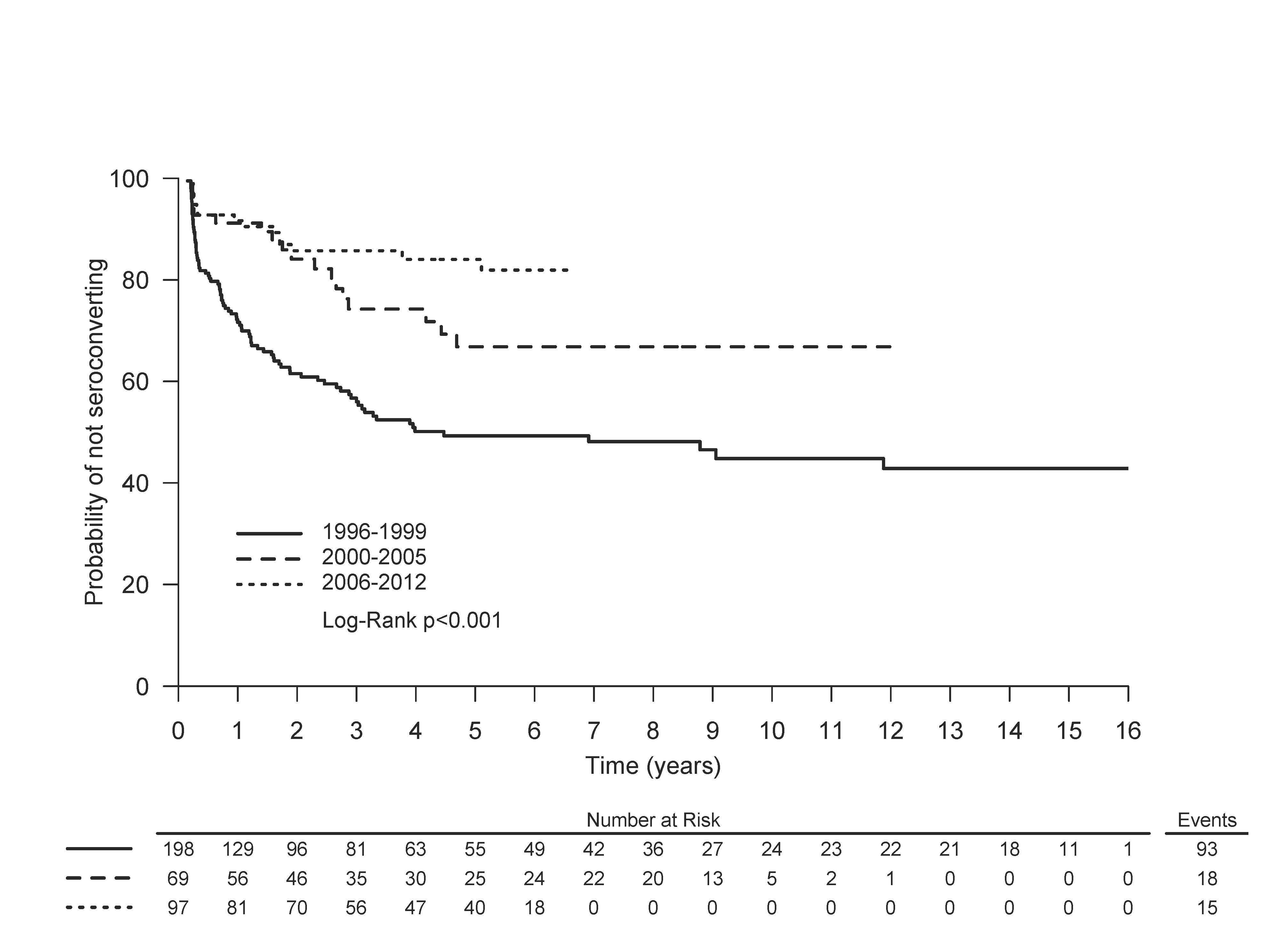

Supplement: Figure S1 — Kaplan-Meier graphs of time to HCV seroconversion by calendar year of enrolment among PWID in the VIDUS cohort between 1996 and 2012 in Vancouver, Canada. (TIF) [file pone.0097726.s001.tif]
