# Supplementary material for: Declining Incidence of Hepatitis C Virus Infection among People Who Inject Drugs in a Canadian Setting, 1996-2012
Source: PLoS One. 2014 Jun 4;9(6):e97726. doi: 10.1371/journal.pone.0097726 (PMC4045728; doi:10.1371/journal.pone.0097726)
Supplement: Table S1 — Participant enrolment characteristics among HCV antibody negative participants with no follow-up visits (1 visit) and follow-up visits (≥2 visits) enrolled in the VIDUS cohort in Vancouver, Canada between 1996 and 2012. (DOCX) [file pone.0097726.s002.docx]

**Supplementary Table S1. Participant enrolment characteristics among HCV antibody negative participants with no follow-up visits (1 visit) and follow-up visits (>2 visits) enrolled in the VIDUS cohort in Vancouver, Canada between 1996 and 2012.**

| Variables | No follow-up visits  (1 visit)  (n=84)  n (%) | Follow-up visits  (>2 visits)  (n=364)  n (%) | *P* |
| --- | --- | --- | --- |
| Median age (25-75^th^ percentiles)* | 25 (21-32) | 27 (22-38) | 0.034 |
| Female sex | 19 (23%) | 115 (32%) | 0.114 |
| High school education or higher* | 33 (40%) | 104 (29%) | 0.064 |
| Unstable housing^†^ | 54 (64%) | 227 (63%) | 0.803 |
| Year of Enrollment |  |  |  |
| 1996-1999 | 55 (65%) | 198 (54%) | 0.163 |
| 2000-2005 | 14 (17%) | 69 (19%) | - |
| 2006-2012 | 15 (18%) | 97 (27%) | - |
| HIV infection^†^ | 9 (11%) | 36 (10%) | 0.841 |
| Crack cocaine use (smoking)^†^ | 43 (51%) | 187 (52%) | 1.000 |
| Syringe borrowing^†^ | 27 (32%) | 96 (26%) | 0.343 |
| Cocaine injecting^†^ | 58 (69%) | 220 (60%) | 0.170 |
| Heroin injecting^†^ | 57 (68%) | 263 (72%) | 0.424 |
| Methamphetamine injecting^†^ | 6 (7%) | 34 (9%) | 0.424 |

Percentages indicate column percentages; *At the time of enrolment; ^†^in the last 6 months prior to enrolment.
